# Efficient Synthesis of a Schiff Base Copper(II) Complex Using a Microfluidic Device

**DOI:** 10.3390/mi14040890

**Published:** 2023-04-21

**Authors:** Masashi Kobayashi, Takashiro Akitsu, Masahiro Furuya, Tetsushi Sekiguchi, Shuichi Shoji, Takashi Tanii, Daiki Tanaka

**Affiliations:** 1 Faculty of Science and Engineering, Waseda University, 3-4-1 Okubo, Shinjuku-ku, Tokyo 169-8555, Japan; shojis@waseda.jp (S.S.); tanii@waseda.jp (T.T.); 2 Department of Chemistry, Faculty of Science, Tokyo University of Science, 1-3 Kagurazaka, Shinjuku-ku, Tokyo 162-8601, Japan; akitsu2@rs.tus.ac.jp; 3 Cooperative Major in Nuclear Energy, Graduate School of Advanced Science and Engineering, Waseda University, 3-4-1, Okubo, Shinjuku-ku, Tokyo 169-8555, Japan; mfuruya@waseda.jp; 4 Research Organization for Nano & Life Innovation, Waseda University, 513 Tsurumakicho, Shinjuku-ku, Tokyo 162-0041, Japan; t.sekiguchi@ruri.waseda.jp (T.S.);

**Keywords:** Schiff base, copper complex, microfluidic, high throughput

## Abstract

The efficient synthesis of amino acid Schiff base copper(II) complexes using a microfluidic device was successfully achieved. Schiff bases and their complexes are remarkable compounds due to their high biological activity and catalytic function. Conventionally, products are synthesized under reaction conditions of 40 °C for 4 h using a beaker-based method. However, in this paper, we propose using a microfluidic channel to enable quasi-instantaneous synthesis at room temperature (23 °C). The products were characterized using UV–Vis, FT–IR, and MS spectroscopy. The efficient generation of compounds using microfluidic channels has the potential to significantly contribute to the efficiency of drug discovery and material development due to high reactivity.

## 1. Introduction

Schiff base ligands have been widely studied for their ease of synthesis using the condensation reaction of primary amines with carbonyl compounds. These ligands can coordinate with most transition metal ions to form complexes. The azomethine nitrogen of Schiff bases is primarily responsible for coordinating with transition metals [1,2]. Schiff bases have attracted a great deal of attention, not only for their application in coordination chemistry but also in medicine and drug discovery. Schiff bases exhibit a variety of biological activities, including antibacterial, anti-inflammatory, and antiviral activities [3,4,5,6,7]. Their biological activities have attracted the attention of researchers worldwide, and their potential as therapeutic agents has been reported in several studies [8,9,10,11,12,13,14].

Among Schiff base complexes, the metal complexes that contain amino acid moieties have garnered significant attention due to their combination of the biologically important features of amino acids that are involved in several biological processes [15,16,17,18]. Amino acids are the building blocks of proteins and play a vital role in metabolic pathways, neurotransmitter transport, and biosynthesis [19,20]. Therefore, the incorporation of amino acids into metal complexes offers a promising avenue for the development of new materials with enhanced biological activity. One of the key advantages of using amino acids in the ligands of metal complexes is their bio-compatibility, which enables them to interact effectively with biological systems. The ability to tune the biological activity of these complexes by modifying the amino acid structure offers a tremendous opportunity for the development of new drugs with high selectivity and specificity. For instance, several studies have reported the potential use of metal complexes containing amino acids as anti-cancer agents due to their ability to target specific cancer cells while leaving healthy cells unaffected [21,22]. The unique properties of these composites are expected to make them attractive materials for biobatteries that can be used as electrode materials, catalysts, and energy storage devices [23].

Chemical synthesis is a crucial step in drug discovery, materials science, and chemical production. To generate new drugs or materials, it is effective to systematically synthesize a large number of chemical species under a large number of conditions [24,25,26]. However, conventional beaker-based synthesis methods are problematic for high throughputs, as each synthesis requires a prolonged amount of time (several minutes or hours). Therefore, chemical synthesis using microfluidic devices is gaining attention in this field. Microfluidic devices are small-scale systems that allow for the precise control of fluids and their reactions [27,28]. Chemical reactions that utilize the diffusion of substances between laminar flows and droplet formation and that occur in microfluidic devices have already been reported [29,30]. These studies have shown that chemical reactions in microfluidic devices have various advantages, such as a high reaction efficiency or the ability to conduct testing with smaller sample volumes [31,32,33]. For example, Meng and colleagues reported that enzymatic reactions using aqueous two-phase systems were 500-times faster than reactions using conventional beakers [34]. This was due to the rapid mixing and diffusion of reactants in the microscale environment. Similarly, Neumann and colleagues showed that synergistic organocatalytic photo redox α-alkylation can achieve yields within 45 min using microfluidic devices that are equivalent to reactions that take 18 h in batch methods [35]. These results demonstrate the potential of microfluidic devices to improve reaction speed and efficiency. High-throughput screening systems that use microfluidic devices are also being developed [36,37]. These systems allow for the parallel synthesis and screening of large numbers of compounds under various conditions. This approach saves time and resources, while increasing the chances of identifying successful compounds. As seen in these papers, miniaturized total analysis systems (μ-TAS) have shown promising results as an alternative to conventional beaker-based chemical synthesis [38].

However, while there has been a great deal of research on the construction of screening systems for drug discovery using droplets prepared in microfluidic channels, there has not been much research on the synthesis of drug candidates in microfluidic channels. Furthermore, in microfluidics, it is important to achieve high throughput by performing all steps on a microchip, and it would not make sense to introduce chemical species prepared in a beaker into a microfluidic device, which is the current method.

This study synthesized copper(II) complexes with Schiff base ligands using a laminar flow microfluidic device. The Schiff base ligands were obtained from isoleucine and salicylaldehyde. The microfluidic apparatus employed in this study was able to synthesize the desired copper(II) complexes stably. The products were characterized using various analytical techniques, such as UV-vis (ultraviolet-visible) spectra, FT-IR (Fourier-transform infrared spectroscopy) spectra, and MS (mass spectroscopy). The results confirmed that the desired amino acid Schiff base copper(II) complexes were successfully synthesized. Furthermore, the reaction took place within 20 s in the flow channel. These results suggest that using microfluidic devices for chemical synthesis may significantly accelerate the drug discovery process by rapidly screening the reaction conditions with minimal reagents. The use of microfluidic devices in chemical synthesis is a valuable tool for researchers involved in drug discovery.

## 2. Results

**Figure 1:**
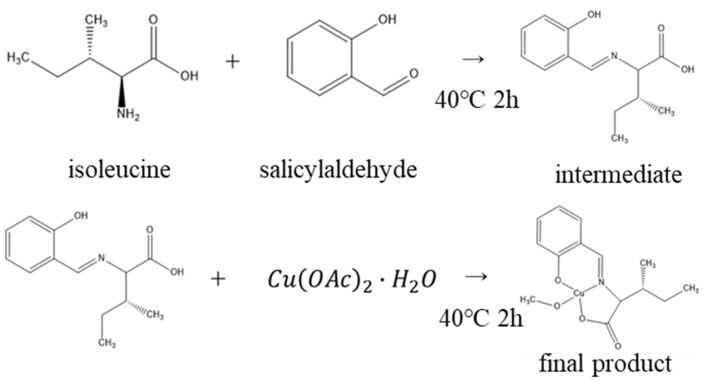
Synthesis reaction of an amino acid Schiff base copper(II) complex.

Figure 1 shows the synthesis reaction of the Schiff base metal complex. Each reagent introduced into the microfluidic device was withdrawn from the outlet and collected through silicone tubing in the sample tube within 20 s. The collected final product was analyzed using UV-vis spectra, IR spectra, and MS. In addition to our microfluidic device, we synthesized identical amino acid compounds using a conventional beaker method as a control sample at 40 °C for 4 h.

### 2.1. UV–Vis Spectrum

**Figure 2:**
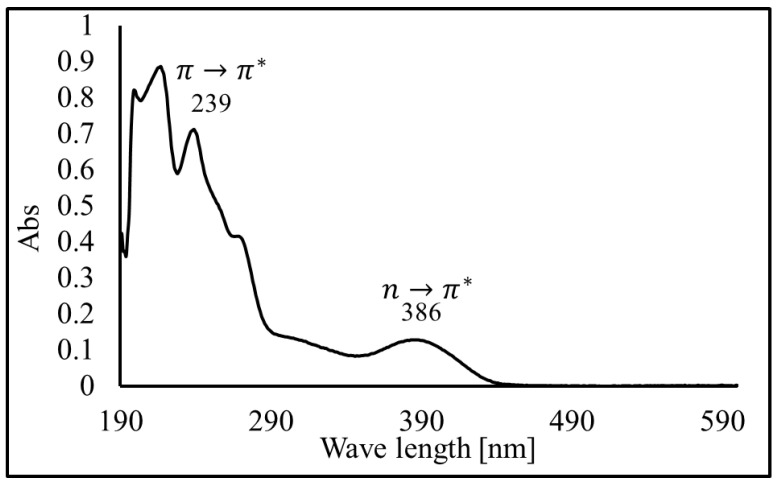
UV–Vis spectrum of an amino acid Schiff base copper(II) complex.

Figure 2 shows the UV–Vis spectrum for the metal complex produced in the microfluidic device. This result confirms the electronic transitions due to the product. The absorption peaks found around 239 nm could be assigned to π–π* transitions of the ligand, and the n–π* transitions at 386 nm might be influenced by the formation of a coordination bond between the ligand and the copper metal ion. This result suggests that the ligand, an amino acid Schiff base, forms a complex with a copper(II) ion.

### 2.2. FT-IR Spectrum

**Figure 3:**
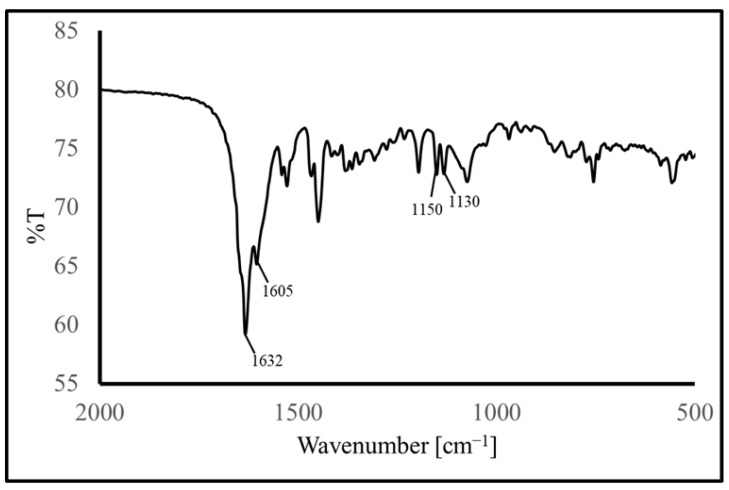
FT–IR spectrum of amino acid Schiff base copper(II) complex.

Table 1 and Figure 3 show the FT–IR spectrum of the synthesized Schiff base copper(II) complex. The intense band at 1632 cm^−1^ was due to the bending vibrations of the C=N bond. The absorption peaks at 1130 cm^−1^ and 1150 cm^−1^ were due to the bending vibrations of the C-O bond. The peak at 1605 cm^−1^ showed the existing vibration of the C=O bond in the ligand.

### 2.3. Mass Spectrum

**Figure 4:**
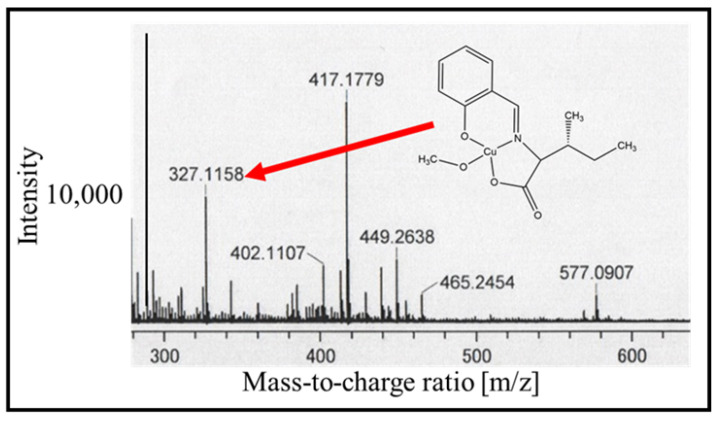
ESI–MS of amino acid Schiff base copper(II) complex.

In Figure 4, the results of MS analysis using the electrospray ionization (ESI) method are shown. The MS measured positive ions. An ion peak with 327 (*m*/*z*), which is assigned to the protonated target copper(II) complex, could be observed.

### 2.4. Reaction Conditions

Table 2 summarizes the advantages of using the microfluidic device to synthesize Schiff base copper(II) complexes. The microfluidic device showed significant improvements in terms of the synthesis time, reaction temperature, and amount of reagents used. While the beaker synthesis required 4 h, the target product was successfully synthesized within 20 s using the microfluidic device. In addition, the reaction conditions of temperature in the beaker experiment were 40 °C on a hot plate. However, in this experiment, the synthesis was successfully carried out at room temperature (23 °C). The sample concentration was 40 mmol/L to enhance reactivity in the beaker, but in the microfluidic device, it was adjusted at 20 mmol/L to prevent the solute from precipitating during the experiment because each reactant needed to be a complete methanol solution. One of the advantages of the microfluidic device, using small amounts of reagents, was also achieved in this experiment. In the beaker, 4 mmol of reagent was used because the reactant was used at a concentration of 40 mmol/L in 100 mL of solution. On the other hand, in the microfluidic device experiment, 10 µmol of each reactant was used per 500 µL syringe of each reaction, which is equivalent to 1/400 of the amount of reagent in a beaker.

## 3. Materials and Methods

### 3.1. Materials

All reagents and solvents were obtained from a commercial supplier and used as received unless otherwise mentioned. L (+)-isoleucine (99.0+%, Titration), salicylaldehyde (97.0+%, mass/mass, C_7_H_6_O_2_, GC), Copper(II) Acetate Monohydrate (98.0+%, Titration), and methyl alcohol (MeOH, anhydrous, 99.8%, mass/mass, C_7_H_6_O_2_, GC) were purchased from FUJIFILM Wako Pure Chemical Corporation (Osaka, Japan).

### 3.2. Chemical Reaction in a Beaker

The synthetic scheme of the chemical reaction used in this study is shown in Figure 1, where the copper(II) complex is carried out in two steps: the preparation of the ligand (Step 1) and the binding of the copper(II) ion (Step 2). The ligand was synthesized using *L*-isoleucine (4 mmol) and salicylaldehyde (4 mmol), which were dissolved in methanol (100 mL) and stirred at 40 °C for 2 h. The metal complex was then synthesized by adding copper(II) acetate dihydrate (4 mmol) to the methanol solution of the ligand and stirring at 40 °C for 2 h. This experiment was conducted in a 200 mL conical beaker. 

### 3.3. UV-Vis Spectrum Analysis

Analysis of the obtained products was carried out using UV–Vis spectroscopy. A UV–Vis spectrophotometer (V-630, JASCO, Tokyo, Japan) was used for the measurement. The resulting product was diluted 10-fold with methanol and in a quartz cell with an optical path length of 10 mm.

### 3.4. FT-IR Spectrum Analysis

IR spectra were measured using an FT/IR-6200 spectrometer (JASCO). The product obtained as a methanol solution was evaporated to reduce solvent, and KBr pellets of the sample were made. The observations were carried out under atmospheric pressure without nitrogen purging.

### 3.5. ESI-MS Analysis

Mass spectrometry was employed for analysis using an LCQ-Fleet ESI-MS spectrometer (Thermo Fisher Scientific, Waltham, MA, USA). Products were diluted 100-fold with methanol and measured. The analysis was performed with positive ion measurements, and the ring lens voltage was set to 40 V.

### 3.6. Fabrication Process of a Microfluidic Device

**Figure 5:**
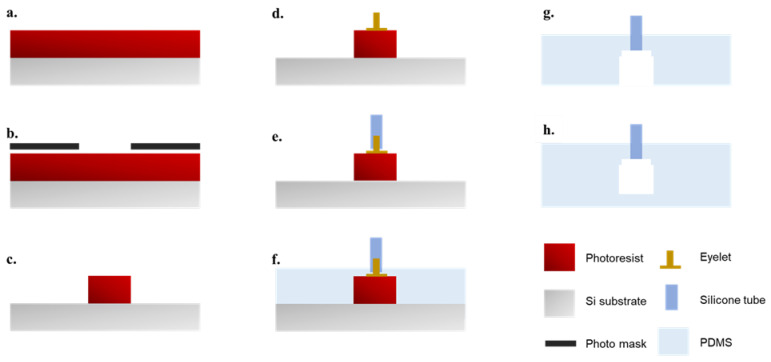
Fabrication process of a microfluidic device. (**a**) Resist coating. (**b**) Exposure. (**c**) Development. (**d**) Bonding eyelet. (**e**) Fabrication of the inlet and outlet section with silicone tube. (**f**) Pouring PDMS into a mold. (**g**) Removing PDMS from the mold. (**h**) Joining of PDMS sheet and channel.

**Figure 6:**
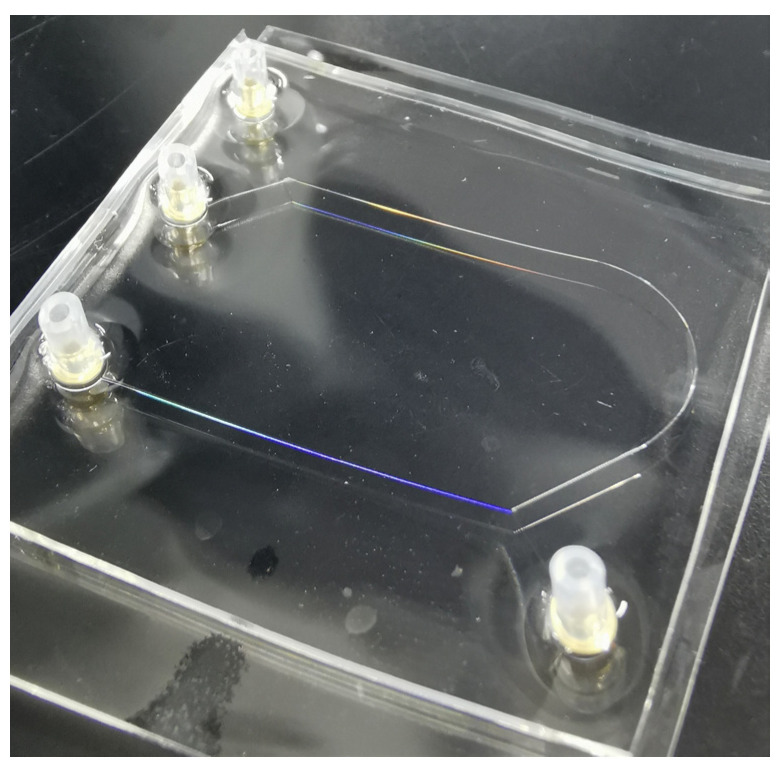
Image of the microfluidic device.

Figure 5 shows the fabrication process for microfluidic devices. First, 100 µm of resist (SU-8 3050, Kayaku Advanced Materials, Tokyo, Japan) was spin-coated onto a 50 mm square Si substrate at 1200 rpm for 30 s, as shown in Figure 5a. The substrate was made by dicing a 6-inch Si substrate (Shin-Etsu Chemical Co., Ltd., Tokyo, Japan). After the resist was applied, the resist-coated Si substrate was heated on a hot plate at 65 °C for 2 min to improve the adhesion between the resist and the substrate and then further heated at 95 °C for 1 h. If the Si substrate is cut to an appropriate size by dicing before this process, it is recommended that ultrasonic cleaning is performed in the order of acetone, IPA, and pure water, because the Si substrate may be contaminated. Figure 5b shows the process of exposing the shape of the channel using a photomask to the resist. The photomask was fabricated by Toyo Seimitsu Kogyo (Tokyo, Japan). After confirming that the resist had cooled sufficiently after heating, exposure was performed with an exposure dose of 250 mJ/cm^2^. The resist was then heated again on a hot plate at 65 °C for 1 min and 95 °C for 5 min, cooled sufficiently, and developed using a developer (SU-8 developer, KAYAKU ADVANCED MATERIALS, Tokyo, Japan). By removing the resist from the unexposed areas, a structure with resist deposited on the Si substrate was obtained, as shown in Figure 5c. The fabrication method of the inlet and outlet of the microfluidic device is shown in Figure 5d,e. Copper eyelets are bonded to the inlet or outlet portion of the channel fabricated with a resist using adhesive. The eyelets have an inner diameter of 1.0 mm, an outer diameter of 1.1 mm, a base of 2 mm, and a height of 3 mm. After confirming that the adhesive had bonded the dovetails to the resist, a silicone tube with an inner diameter of 1 mm and an outer diameter of 2 mm was inserted into the eyelets. At this time, we were careful not to insert the silicone tube completely, as it needs to be removed from the eyelets. Figure 5f–h show the procedure for fabricating the polydimethylsiloxane (PDMS; SILPOT 184 W/C, Dow Chemical Japan Ltd., Tokyo, Japan) channels. PDMS was thoroughly mixed with the curing agent in a 10:1 ratio and placed in a vacuum vessel at 0.1 MPa to remove air bubbles until they were completely eliminated. The PDMS was then poured into the mold made in Figure 5e up to the height of the silicone tube and heated at 120 °C for 30 min. At the same time, the PDMS base for the bottom of the microfluidic device was also prepared. After heating, the PDMS was carefully cut out of the mold using a cutter. The cut PDMS was then fully hydrophilized using O_2_ plasma and laminated to a flat PDMS plate at the bottom. The microfluidic device was fabricated by heating the PDMS substrates at 120 °C for 2 h to strengthen the adhesion between the PDMS substrates. The image of the device used for synthesizing the Schiff base copper(II) complex is shown in Figure 6.

### 3.7. Synthesis Method in a Microfluidic Device

**Figure 7:**
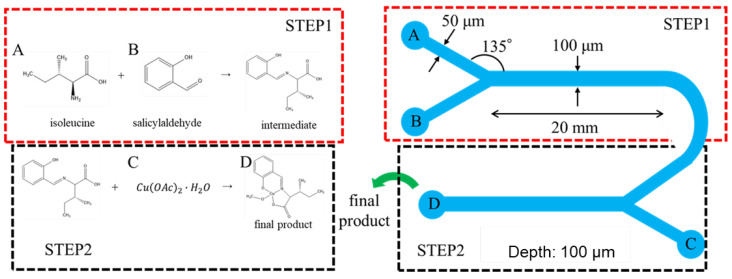
Synthesis flow in a microfluidic device.

Figure 7 illustrates the reaction mechanism of the two-step microfluidic synthesis of a Schiff base copper(II) complex. The reactions were conducted in two different Y-shaped microfluidic devices, where isoleucine and salicylaldehyde were introduced into the inlets A and B, respectively, with a flow rate of 5 µL/min. The top part of the device, referred to as STEP1, was utilized for the synthesis of the Schiff base ligand. In the bottom part, STEP2, a solution of copper(II) acetate dihydrate (20 mmol/L) was induced through inlet C with a flow rate of 10 µL/min. The ligand of the copper(II) complex was synthesized and extracted from outlet D as a solution. The reactants were dissolved in methanol and introduced into the inlets using syringes (1750CX, Hamilton, Reno, NV, USA) and syringe pumps (Le-gato 111, KD Scientific, Holliston, MA, USA). This allowed for precise control over the flow rate and the amount of reactants introduced into the microfluidic device.

### 3.8. Device Design

In this experiment, a simple laminar flow microfluidic device with a Y-shaped structure was utilized. The experiment was designed to enable the consecutive synthesis of the Schiff base ligand and its subsequent complexation with copper(II) ions, as shown in Figure 1. Two Y-shaped channels were prepared and arranged in series for each reaction, as depicted in Figure 7, to allow for the formation of the copper(II) complex with the ligand. Our focus in this experiment was to synthesize significant chemical reactions that have potential applications in drug discovery and material development using a novel approach involving a microfluidic device that provides ease of operation, high reproducibility, and stability. Therefore, we did not discuss optimization methods, such as introducing a mixing structure into the microchannel to generate turbulent flow in the laminar flow device, which has been previously studied, or implementing more quantitative reaction conditions using microdroplets [39,40,41,42]. 

## 4. Conclusions

An amino acid Schiff base copper(II) complex was synthesized in 20 s without temperature control for the first time using a microfluidic device. The experiment confirmed that the reaction efficiency was about 700-times higher than that of synthesis using a beaker, focusing on the time required for synthesis. Therefore, we believe that the synthesis of Schiff base copper complexes in a short time will contribute to high throughput in drug discovery and materials development.

## Figures and Tables

**Table 1:** The results of IR spectrum analysis.

IR Bands	Observed (cm^−1^)	Expected (cm^−1^)
C=N	1632	1615–1700
C-O	1130, 1150	1100–1300
C=O	1605	1550–1610

**Table 2:** Comparison of reaction conditions between beakers and the microfluidic device.

	Beaker	Microfluidic Device
Total reaction time	4 h	~20 s
Temperature	40 °C	23 °C
Reagent concentration	40 mmol/L	20 mmol/L
Reagent Volume	4 mmol	10 µmol

## Data Availability

Not applicable.
